# Feasibility of a randomised controlled trial of Healthier Wealthier Families in Sweden: results from the Ameliorating Child poverty through Connecting Economic Services with child health Services (ACCESS) pilot study

**DOI:** 10.1186/s12913-025-13753-y

**Published:** 2025-12-01

**Authors:** Nina Johansson, Anna Sarkadi, David Isaksson, Georgina Warner

**Affiliations:** 1 Child Health and Parenting (CHAP), Department of Public Health and Caring Science, Uppsala University, Uppsala, Sweden; 2 Health Services Research, Department of Public Health and Caring Science, Uppsala University, Uppsala, Sweden

**Keywords:** Child poverty, Economic services, Child health services, Healthier Wealthier Families, Sweden, Randomised controlled trial, Internal pilot

## Abstract

Despite universal measures in Sweden, the proportion of children living in economic hardship is increasing. One strategy to mitigate hardship is integrating financial counselling within healthcare services. This paper describes a pilot randomised controlled trial (RCT) of the ‘Healthier Wealthier Families’ model in which child healthcare nurses screen parents of young children for economic hardship and refer those in need to free budget and debt counselling (BDC). The study was an internal pilot of a two-arm randomised waitlist-control trial with a one-to-one allocation. The intervention arm received BDC immediately after randomisation, while the waitlist-control arm was referred 3 months later. Both arms received a financial guidance book. Assessments were conducted at two points: pre-intervention (T1) and post-intervention (T2; 3 months post-randomisation). The main objective was to assess the feasibility of recruitment, randomisation, data collection, attendance, and adherence to the topic guide. Of 368 screened parents, 111 (30.2%) were eligible, most of whom were identified by two of the five screening questions focused on ‘worrying about running out of money’ and ‘meeting an unexpected cost’. The conversion rate from screening positive to consenting for trial was 18.0% (n = 20). Reasons for declining were due to stigma, lack of capacity or already available knowledge and support. The randomisation was effective, with equal distribution between trial arms. No contamination was detected. Eight out of ten participants who were allocated to the intervention attended at least one session; the mean number of sessions was two. Topic guide implementation was inconsistent. There were high completion rates for most measures, except for income. The primary outcome measure of Child Material and Social Deprivation showed a floor effect, with many parents reporting zero ‘enforced lack’ items. Results from the pilot RCT indicate that the screening process identified the anticipated proportion of eligible parents (30.2%), but that three of the screening questions are redundant. The conversion rate was much lower than anticipated, questioning the feasibility of a full-scale RCT. BDC fidelity to protocol could be strengthened with implementation support. Finally, the primary outcome measure should be reconsidered, as well as the way in which participant income is captured.

**Supplementary Information:**

The online version contains supplementary material available at 10.1186/s12913-025-13753-y.

## Trial registration

Clinicaltrials.gov; NCT05511961. Prospectively registered on 23 August 2022. https://clinicaltrials.gov/ct2/show/NCT05511961.

## Background

Poverty and deprivation can be understood as limited resources in relation to the needs of the individual [1]. The implications of child poverty are well-researched in terms of social determinants of health [2], mental health [3], family stress [4] and child development [5]. There is evidence of poverty being related to perceptions of stigma, and its negative effect on health [6, 7]. Over the last decades, there has been an increase in financial inequality [8], especially evident in the aftermath of the COVID-19 pandemic [9, 10]. It is not an issue confined to developing countries; in rich democracies, like Sweden, child poverty and economic hardship are increasingly prevalent [11].

A common indicator of economic hardship in Sweden is the inability to face unexpected expenses. In 2023, 20.3% of all Swedish households could not meet an unexpected expense of 13,000 SEK (approx. £950) [12]. Another measurement is relative poverty, which shows the proportion of households living with a disposable income below 60.0% of the national median yearly income, which was 450,800 SEK (approx. £30,700) in 2023 [13]. The latest statistics show that 17.0% of children in Sweden live in households experiencing relative poverty [14]. A third measure is ‘Material and Social Deprivation’ (MSD), which captures how living conditions can be negatively affected by economic hardship [15]. While Swedish MSD is considered low in relation to other EU countries [16], in 2023 there was a high discrepancy in MSD between two-parent households (4.6%) and single parent-households (13.6%) [17].

Despite the universal measures being used across Nordic countries to reduce poverty and provide social support, inequalities are prevalent [18]. With increasing disparities, there is a need for a policy shift [19] and universal actions that are proportionate to the level of disadvantage [2]. One way to ameliorate hardship and poverty can be through linking general health care with financial support services [20–23]. The Healthier Wealthier Children (HWC) project, funded by the Scottish Government, set up pathways for referrals between the Scottish National Health Service for young children and the local financial counselling services [20]. The HWC project entailed 2,516 referrals to the counselling service, out of which 54.0% accessed the service to some extent [24]. The quantitative results of the project demonstrated an overall financial gain of £3 million, with an average cliental financial gain of £3.404. Qualitative interviews with participants (*N* = 12) receiving financial counselling, showed improved health and well-being, including reduced stress levels and strengthened family relations. HWC is today included in the Scottish Government policy and has spread to international contexts. The model was later adapted to an Australian context, then referred to as the Healthier Wealthier Families (HWF) project, which aimed to test the feasibility of setting up and evaluating referral pathways between universal health services and financial counselling through a pilot randomised controlled trial (RCT) [25]. The trial faced recruitment challenges, with only thirteen parents recruited. It coincided with the COVID-19 pandemic, which may have affected recruitment; however, qualitative interviews with participating professionals (*N* = 15) revealed that stigma, limited knowledge of financial counselling, a lack of financial literacy, and research burden may have also contributed to the recruitment issues.

In Sweden, the universal child health care system (CHS) is free of charge, covers the vast majority of the child population and is available nationwide [26]. The nurses at CHS receive specialist training and routinely meet families from newborn to 5 years of age, with regular visits in the beginning that become less frequent as the child gets older. The routine visits cover topics such as weight and height development, breastfeeding, dental care and psychosocial factors [26]. Budget and debt counselling (BDC) is a freely available state-run service, legislated to be offered in all 290 municipalities in Sweden [27]. The services are managed by the Swedish Consumer Agency (SCA) and offer counselling in handling debts, managing money and consumer rights [28, 29]. The services are not obliged, yet encouraged, to work preventively.

When introduced to a Swedish setting in 2020, an adaptation of the HWF model was conducted together with representatives from Swedish CHS and BDC [22]. An initial qualitative evaluation of the model in a single municipality explored the perceptions and experiences of the model in a Swedish context, to inform future scale-up. Similar to the Australian HWF evaluation, some implementation obstacles were described. Interviews with the participating nurses (*N* = 7) and budget- and debt counsellors (*N* = 5), as well as parents who declined (*N* = 10) and received (*N* = 9) financial counselling, described how the impacts of the COVID-19 pandemic, high workloads, ambiguities regarding professional roles and stigma all potentially affected the implementation of the model. However, there was an identified importance of supporting families with small children living in economic hardship using the CHS as a point of entry [22]. Therefore, the decision was taken to scale HWF to other municipalities and evaluate the impact of the model.

This paper reports on the continued work of testing HWF in Sweden, and specifically on the outcome of an internal pilot study of the Ameliorating Child poverty through Connecting Economic Services with child health Services (ACCESS) randomised controlled trial (RCT) [30]. The ACCESS trial was designed as a waitlist-control trial, with the intervention arm being referred to BDC immediately and the control arm being referred around 3 months after randomisation. Full details of the ACCESS trial design can be found in the protocol paper [30]. As per good practice recommendations [31], the primary objective of the internal pilot study was to assess the feasibility of the RCT processes. We sought to determine the feasibility of recruitment, randomisation procedure, and data collection, as well as BDC attendance, adherence to the HWF BDC topic guide, and appropriateness of the primary outcome measure. The pilot phase of the trial was defined by the number of participants, with the first twenty parents randomised to the ACCESS trial constituting the internal pilot study sample. Once this recruitment target had been achieved, the feasibility of the RCT processes was assessed to inform future RCT conduct.

## Objectives

The objectives of the study were to:

i. Determine screening and randomisation conversation rates;
ii. Assess the feasibility of randomisation;
iii. Monitor the implementation of BDC; and.
iv. Assess the feasibility of outcome measurement.

## Methods

### Trial design

The study was planned as an internal pilot of the ACCESS trial (Johansson et al., 2022), a two-arm randomised waitlist-control superiority trial with a one-to-one allocation ratio. The intervention arm was referred to BDC immediately after randomisation and the waitlist-control arm 3 months later. Upon screening, all participants were offered a copy of the material ‘Your child & your money’, a financial guidance book developed by the Swedish Financial Supervisory Authority targeting new parents. RCT assessments took place at two points: pre-intervention (T1) and post-intervention (T2; 3 months after randomisation). Due to BDC being a universal and freely available service, processes were in place to check for trial arm contamination. The T2 assessment commenced with an inquiry regarding whether the participant had met with a financial counsellor. In addition, upon control arm referral, BDC checked their records to see if the participant had previously accessed the service. The stepped rules of thumb outlined by Bell et al. [32] were consulted to determine the sample size for the pilot study. Given that the main trial was powered at 80% to detect a medium effect size with *N* = 71 per arm, the pilot study was planned with *N* = 10 per arm [32]. This was considered sufficient given that the purpose of the pilot study was to test the feasibility of RCT processes, not to estimate the intervention effect size or variability of measures.

Ethical approval for the study was obtained from the Swedish Ethical Review Board (Ref. 2022–01391-01) on 14th April 2022 and the trial was prospectively registered at Clinicaltrials.gov (NCT05511961) on 23rd August 2022. The study protocol was developed by an interdisciplinary research team, with patient and public involvement (PPI) from a group of parents with lived experience of financial hardship, as well as CHS nurses, BDC counsellors, social workers and Swedish and international researchers.

### Setting and recruitment

Sweden is divided into 21 regions and 290 municipalities [33]. Besides being geographical areas, regions and municipalities are public governance actors where the regions mainly manage healthcare, such as the CHS centres, and municipalities handle social services, such as the BDC services [34]. Sweden is a large country in relation to its 10.5 million inhabitants, where the largest municipality hosts close to a million inhabitants, while the smallest municipality hosts 2,500 inhabitants [35]. Municipalities are divided into residential areas, where citizen-rich municipalities can have a large number of areas with local CHS centres, and smaller municipalities have fewer residential areas. This can be seen in Table 4, which showcases the population differences in the Swedish settings. In this study, the term ‘site’ refers to the local collaboration setting, consisting of CHS and BDC services within the same municipality or residential area.

Site recruitment was conducted over a time period of two years, and information about the recruitment process was saved continuously in the form of field notes. Due to the region’s responsibility for healthcare, the site recruitment process was initiated at the regional level. All 21 regions were initially considered, yet three were excluded due to other trial engagements. Therefore, 18 out of the 21 regions were approached. In the end, 5 regions were included in the trial. The recruitment was based on the proportion of relative child poverty at the municipal or area level, where CHS centres with a relative child poverty above 19.7% were targeted for participation in the RCT. In the included sites, the relative poverty rate ranged from 20.0 to 41.0%, see Table 1. Sites 1 and 2 are services located in residential areas within two large municipalities, sites 3 and 4 are services within the same municipality, and sites 5–7 are services in smaller municipalities.

**Table 1:** Site characteristics

Site	Relative poverty^a^ (%)	Residential area population^b^ (*N*)	Municipality population^b^ (*N*)	Children 0–4 years^b, c^ (*N*)
1	20	3,640	604,325	186
2	29	6,561	361,974	242
3	22	1,722	28,325	92
4	41	2,054	28,325	85
5	26	24,654	24,665	1184
6	24	16,458	16,482	990
7	35	10,034	10,058	434

^a^ Official Statistics of Sweden, economic standard data for 2023 [14] ^b^ Official Statistics of Sweden, population data for 2023 [36] ^c^ Statistics on area level only available for children 0–4 years, RCT target population 0–5 years

### Participants

The target population was parents with children aged 0–5 years in contact with CHS. To meet the inclusion criteria for participation, parents needed to self-report at least one item indicating economic hardship (see Table 2) and have not been in contact with a financial advisory service in the last month. The screening questions were adopted from the Australian model [37] and adapted to a Swedish setting. When identified as eligible and interested in participation, parents were provided with the study information by the CHS nurse. Informed consent was collected by the first author (NJ) before the pre-intervention assessment. The CHS meet families for routine visits from newborn to the age of 5 years. The CHS services were given the mandate to decide at which of the routine visit(s) the screening would take place. The majority opted to target the 8-month visit, yet there were some variations in targeting earlier or later visits.

### Randomisation

Participants were randomised 1:1, direct referral to BDC (intervention) or referral to BDC after 3 months (waitlist-control) by the first author (NJ) using an online randomisation software application (sealedenvelope.com) set up by the last author (GW). Participants were informed of the randomisation outcome by the first author (NJ). Seven randomisation lists were created, one for each CHS site involved in the pilot phase of the trial. As set out in the study protocol (Johansson et al., 2022), the internal pilot phase continued until 20 eligible families (10 per arm) were randomised.

### Intervention

The intervention arm was referred to BDC immediately after randomisation. A topic guide on how to work preventatively with families was co-developed with BDC counsellors and parents with lived experience of economic hardship and embedded in the usual service setting. The guide, arranged across three sessions, prompted discussion on needs, personal goals, financial behaviour and income maximisation. A fidelity checklist was developed to capture which topics were covered during the sessions. Participants were able to end counselling at any time, and there were no restrictions on access to other support during the study period.

### Data collection

A survey questionnaire developed for the current study was used in the data collection. Measurements of trial outcomes were administered pre-intervention (T1) and 3 months after randomisation (T2). Data were collected using the Research Electronic Data Capture (REDCap) tool hosted at Uppsala University [38, 39]. REDCap is a secure, General Data Protection Regulation (GDPR) certified, web-based software platform designed to support data capture for research studies. Participants were able to complete the survey in their language of choice (Swedish, English, Arabic, Tigrinya, Dari or Somali), with interpreters made available by phone to give extra language support if necessary. To compensate for their time, participants were offered a shopping voucher valued at 200 SEK (approx. £15) on each assessment occasion. Full details of the outcome measures have been published in the trial protocol (Johansson et al., 2022).

### Assessment of pilot study outcomes

Field notes of the site recruitment process were assessed. Screening and conversion rates were determined by the screening survey, completed by nurses, and trial management records kept by the research team. Reasons for declining participation were analysed using content analysis [40]. The feasibility of randomisation was determined by trial management records, participant demographic data derived from the parental pre-intervention survey and the fidelity checklists, completed by BDC counsellors. An assessment of trial arm contamination was conducted by cross-checking participant Swedish ID numbers with BDC service records. Counselling attendance and topic guide fidelity were determined by fidelity checklists, completed by BDC counsellors. The feasibility of outcome measures was determined by the pre-intervention and 3-month follow-up surveys, completed by parents.

The research team utilised the A process for Decision-making after Pilot and feasibility Trials (ADePT) framework [41] when making recommendations based on the pilot trial findings. This framework offers a structured approach to identify and evaluate solutions for challenges encountered in pilot trials. The initial step involves categorising the issues into three types: Type A, which are problems expected to affect only the trial; Type B, which could pose problems for both the trial and real-world settings; and Type C, which are likely to be relevant solely in the real world. The next step is to explore potential solutions by examining which elements of the trial design or the real-world setting may be modified.

### Data analysis

Given this was a pilot study with a small sample size, the analyses of the outcome measures were exploratory and descriptive. We present the data using means and standard deviations for continuous measures, and frequencies and percentages for categorical measures. The data were analysed in R Statistical Software (R version 4.4.1).

## Results

### Feasibility of site recruitment

The intention of this trial was to continue testing the HWF model in Swedish settings of low economic standard. Out of the 18 approached regions, 6 declined with reference to another regional project, and 4 declined due to not having the capacity or need for the model. Among the 8 regions expressing interest, CHS and BDC services in 40 sites were approached. The field notes showed a difficulty aligning interest and capacity among the two different services. In the end, 7 sites across 5 regions were recruited, with the first site commencing in September 2022 and the final site in January 2024.

### Screening and conversion rates

**Fig. 1:**
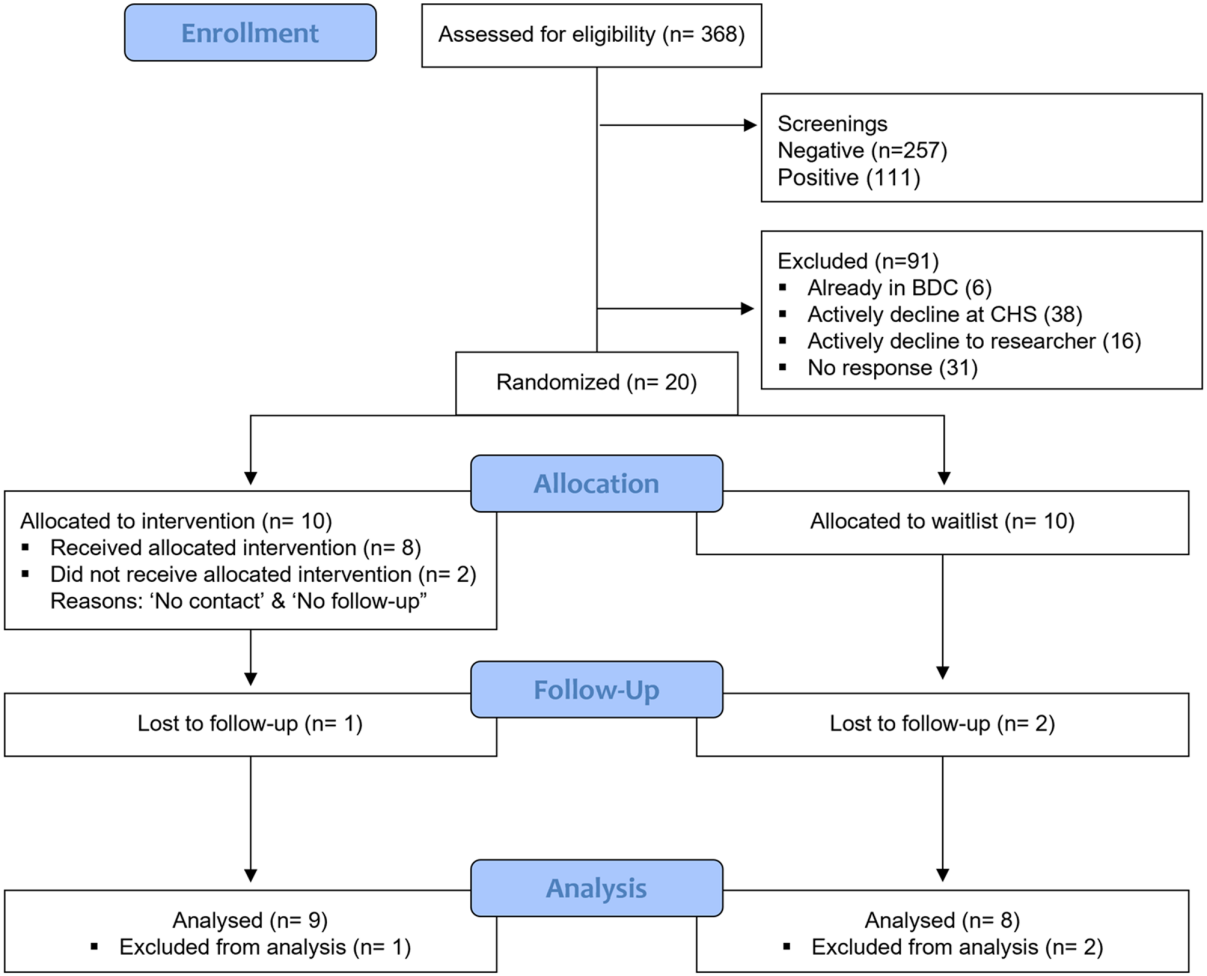
Consort flow diagram

The total sample of individuals approached about the study was 388, of which 368 (94.8%) consented to be screened for eligibility, see Fig. 1.

**Fig. 2:**
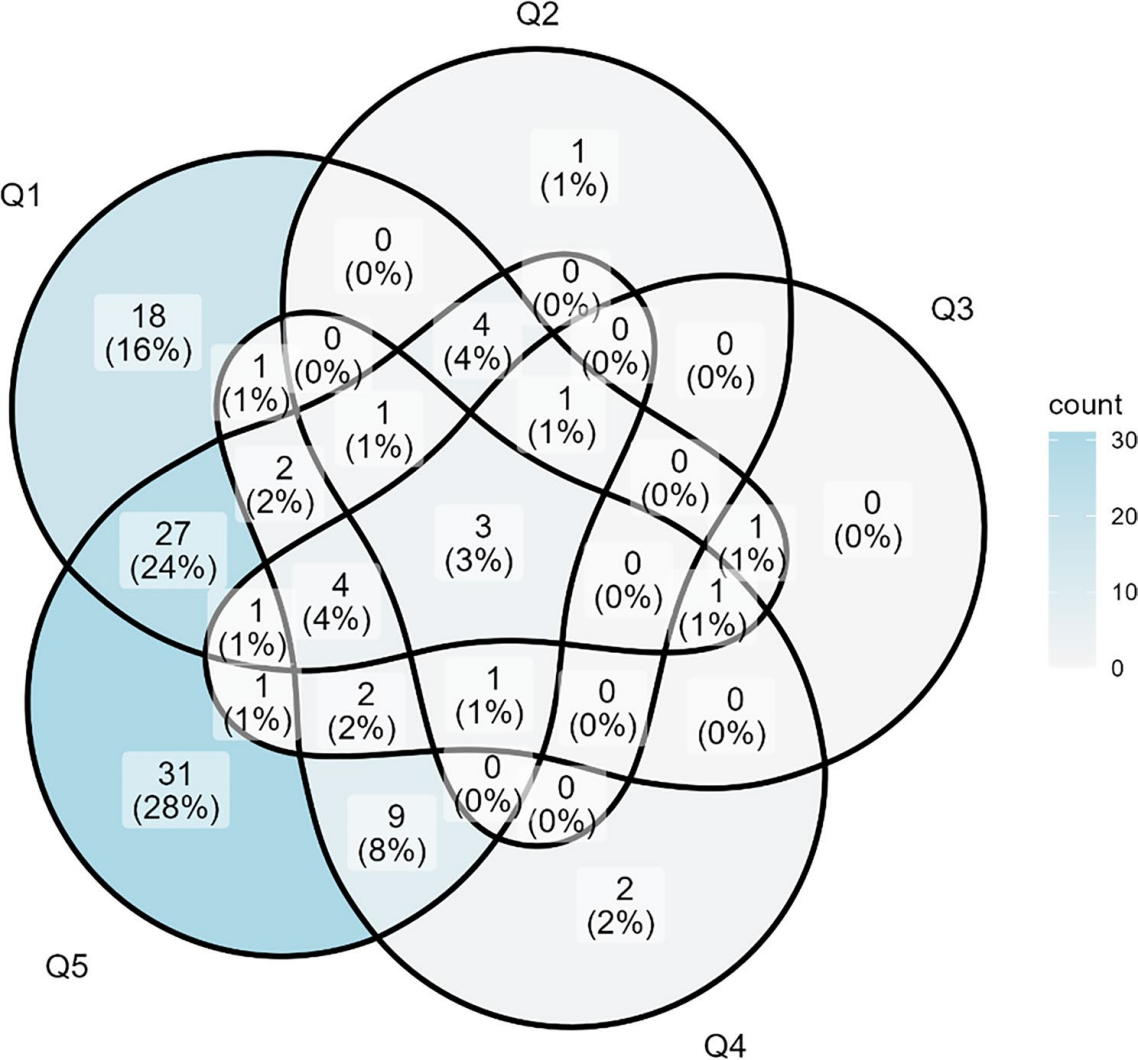
Venn diagram of the distribution of positive responses across screening questions

Positive screening outcomes per question are presented in Table 2, and the distribution across the questions is presented in Fig. 2. The findings showed that ‘worrying that the family might run out of money by the end of the month’ and ‘being unable to handle an unexpected expense of 20,000 SEK (approx. £1,500)’ were the primary screening outcomes.

**Table 2:** Positive screening outcomes, *N* = 111

Screening item	*n*
Q1. Worried family will run out of money by end of month	64
Q2. Unable to pay current expenses such as rent, bills, insurance	11
Q3. Unable to buy necessary items such as clothes and food for yourself and your children	15
Q4. Nobody in the household with a paid job	26
Q5. Unable to handle an unexpected expense of 20,000 SEK (approx. £1,500)	87

Out of the 38 parents who actively declined study participation at the CHS, 35 motivated their answer. Content analysis brought forward two main categories and three sub-categories, see Table 3. In the first category of external factors, the eligible parents either motivated their decline with reference to available support from family and friends, i.e., ‘A network to fall back on’, or considered their economic hardship to be transient due to factors such as parental leave, i.e., ‘Just worrying times’. In the second category of internal factors, some participants expressed that counselling was unnecessary as they already felt financially literate, i.e., ‘Know-how is not the problem’. Others expressed annoyance, classified as the sub-category ‘Mind your own business’, which was interpreted as an expression of stigma related to economic hardship and the prestige of handling it personally. The final sub-category ‘Too much stuff, too little time’ pinpoints the reality for parents to juggle everyday life while managing challenges like financial restraints.

**Table 3:** Reasons given for declining participation in the study

Theme	Category	Sub-category	Example statements
Thank you but no thank you	External factors	A network to fall back on	The person has no need or interest. The eldest son has a job and helps out when needed.
		Just worrying times	We’re not really that worried, just a bit concerned right now, but nothing serious.
	Internal factors	Know-how is not the problem	Parents consider themselves to be financially literate and able to manage the situation.
		Mind your own business	The father was annoyed: ‘That’s our business’.
		Too much stuff, too little time	Being a single mother of four, while studying. There is no time.

A further 47 participants were lost after screening, but prior to pre-intervention assessment. Of these, 16 indicated to the researcher that they did not wish to participate in the study, and 31 did not respond despite repeated invitations. It might be the case that the burden of having to fill in a questionnaire and the prospect of being randomised caused these parents to decline, despite being initially positive to being screened and having their contact details shared with the researchers for inclusion in the trial.

### Sample

Characteristics of the included study sample are presented in Table 4.

**Table 4:** Participant characteristics at T1

	Intervention (*N* = 10)		Waitlist (*N* = 10)	
	**Mean**	**SD**	**Mean**	**SD**
**Age**	32.9	6.2	32.3	6.2
**Number of children**	1.8	1.1	2.2	1.0
	**N**	**%**	**N**	**%**
**Gender**				
Female	8	80	7	70
Male	2	20	3	30
**Occupation**				
Employment	7	70	4	40
Self-employment	-	-	1	10
Unemployment	2	20	1	10
Other	1	10	4	40
**Civil status**				
Partner	7	70	7	70
No partner	3	30	3	30
**If partner**				
Shared household	7	100	7	100
**Housing**				
First-hand rental contract	6	60	8	80
Sublease rental (third party etc.)	-	-	-	-
Residential apartment	1	10	1	10
Homeowners	3	30	1	10
**Educational level**				
Primary school or equivalent	2	20	1	10
Secondary school diploma or equivalent	4	40	6	60
University education of less than 3 years	2	20	2	20
University education of 3 years or more	1	10	1	10
Other	1	10	-	-
**Origin**				
Sweden	7	70	6	60
In a European country outside the Nordic region	1	10	-	-
In non-European country	2	20	4	40
**Language spoken at home**				
Swedish	6	60	6	60
Other	3	30	4	40
Missing	1	10	-	-
**Age of children**				
Under 1 year	10	55.5	6	27.2
1–2	4	22.2	7	31.8
3–5	2	11.1	3	13.6
Older than 5	2	11.1	6	27.2
**Gross Household Income**				
Lower than 20 000	3	30	1	10
20 000–29 999	3	30	4	40
Higher than 30 000	4	40	3	30
Missing	-	-	1	10
**Fixed Household expenses**				
Lower than 15 000	3	30	6	60
15 000–25 000	3	30	2	20
Higher than 25 000	2	20	1	10
Do not know	1	10	-	-
Missing	1	10	1	10

### Feasibility of randomisation

The outcome of the 20 randomisations was evenly distributed across the intervention (*n* = 10) and waitlist-control (*n* = 10) arms. There were no reported issues with the randomisation website (www.sealedenvelope.com). No trial arm contamination was detected.

### Counselling attendance and topic guide fidelity

Out of the eight intervention arm participants attending at least one financial counselling session, six were registered in the intervention fidelity checklist. BDC counsellors reported that the remaining two participants were not registered as the contact with the client was considered a ‘short, general talk’ conducted over the phone. The average number of counselling sessions was 2, ranging from 1 to 4 sessions and the average session time was 31 min, with a range of 10 to 60 min.

In terms of the fidelity checklist, for the first counselling sessions, all sessions covered the topics of why the clients were seeking counselling and their goal(s). Five out of six sessions covered ‘income sources’, while ‘money management’ was covered in three sessions. The counsellor and client decided on a ‘task for the next meeting’ in three of the sessions. A ‘review of completed tasks’ was performed with two of the clients in the second session; however, the third client did not attend the second session. In total, four clients attended a second session. During these sessions, ‘financial planning’ and ‘money management’ were covered in two sessions each. Only one client attended a third and fourth counselling session, during which the topics of ‘money management’ and ‘sources of income’ were covered.

### Feasibility of outcome measurement

Completion rates, presenting the proportion of participants answering the whole survey questionnaire, were high for the majority of topics in the survey, both at T1 and T2 (see Table 5).

**Table 5:** Completion rates in %, [CI high, CI low], T1 *N* = 20, T2 *N* = 17

	*n*	T1	95% CI	*n*	T2	95% CI
Child MSD	18	90.0	[68.3–98.7]	15	88.2	[63.5–98.5]
Financial control	19	95.0	[75.1–99.8]	17	100.0	[80.4–100.0]
Stigma	19	95.0	[75.1–99.8]	15	88.2	[63.5–98.5]
GHQ12	19	95.0	[75.1–99.8]	16	94.1	[71.3–99.8]
Financial knowledge	19	95.0	[75.1–99.8]	16	94.1	[71.3–99.8]
Readiness for change	20	100.0	[83.1–100.0]	17	100.0	[80.4–100.0]
Capability	18	90.0	[68.3–98.7]	17	100.0	[80.4–100.0]
Municipal intervention	12	60.0	[36.0-80.8]	11	64.7	[38.3–85.7]
Household income	18	90.0	[68.3–98.7]	15	88.2	[63.5–98.5]

For the survey questions regarding sources of income, 100.0% [83.1, 100.0] and 100.0% [ 80.4, 100.0] responded to at least one alternative to income source at T1 and T2, respectively. No participant responded to the whole questionnaire regarding the consumption of resources. On the question regarding setting a goal for the counselling, 16 out of 23 participants responded at T1, and 16 followed up their response at T2.

The median completion time for the T1 survey was 26 min (Missing 4), with four outliers taking over 24 h, for whom it is assumed the survey link was left open and returned to at a later stage. For T2, the average completion time was 31 min (Missing 4), with two outliers. The T2 survey was distributed 3 months after the participant’s randomisation date, and the time period for completion ranged from 93 to 151 days. The T2 survey was completed by 9 respondents in the intervention arm and 8 respondents in the waitlist-control arm.

### Descriptive statistics of outcome measures

Table 6 provides an overview of the average scores on the primary and secondary outcome measures, i.e., Child MSD, financial control, perceived financial stigma and parental mental health (GHQ-12), at T1 and T2. The majority (75.0%) of respondents indicated zero ‘enforced lack’ items on the Child MSD at T1. Financial control appeared to change in both arms, with little variation in SD. The reported levels of perceived financial stigma were higher for both arms at T2, with little variation in SD. Parental mental health appeared to go down for the intervention arm at T2, while it improved in the waitlist-control arm; yet, greater variation was seen in the latter arm. In the intervention arm, the mean rating of whether personal goals had been achieved at T2, on a scale of 0–10, was 5.9 (SD 3.9).

**Table 6:** Summary of findings at T1 and T2 for the intervention arm and waitlist-control armpresented as mean (SD) [95% CI]

	Intervention		Waitlist-control	
Variable	T1 (*n* = 10)	T2 (*n* = 9)	T1 (*n* = 10)	T2 (*n* = 8)
Child MSD	5 (2.8) [1.0-8.9]	2.6 (2.0) [0.3-5.0]	3.1 (2.4) [1.2-5.0]	2.2 (1.5) [0.7–3.7]
Financial control	3.0 (0.6) [2.6–3.4]	3.1 (0.6) [2.6–3.5]	3.2 (0.6) [2.7–3.7]	3.4 (0.8) [2.7–4.1]
Perceived financial stigma	2.3 (1.2) [1.4–3.1]	2.5 (1.1) [1.6–3.4]	2.1 (0.4) [1.8–2.4]	2.45 (0.4) [2.0-2.8]
Parental mental health (GHQ-12)	14.1 (7.2) [8.9–19.2]	14.5 (6.1) [9.8–19.2]	14.1 (5.4) [10.2–18.0]	12.07 (7.7) [5.5–18.5]
Income below 21,150 SEK*	*N* = 2	*N* = 0	*N* = 1	*N* = 0

*Swedish relative poverty level

## Discussion

The purpose of this study was to pilot the feasibility of an RCT of the HWF model in Sweden, to inform a future full-scale trial. The findings presented certain aspects of a successful trial but overall, a design unfit for a large-scale RCT. Presented strengths were screening rates aligned with preliminary predictions, the randomisation process resulting in evenly distributed participants, no detected trial arm contamination, and a good survey response rate. The result showed ‘Type A’ problems indicating problems with the trial, e.g., the primary outcome measure was deemed unfit due to floor effects. The result also showed ‘Type B’ problems, indicating problems with both the trial and the context, exemplified in the conversion rate being much lower than anticipated, questioning the feasibility of a full-scale RCT.

### The snakes and ladders of site recruitment

Recruitment was a major issue in this trial, initially identified when trying to establish contact with the Swedish regions. The main reasons for declining were other ongoing or planned trials in the region. In the next step, there was a challenge in aligning service engagement. Although all the approached CHS service personnel agreed with the presented literature demonstrating how finances affect family health and child development [4, 5], a substantial portion of those who were informed of the study were reluctant to participate. Similar to the findings of HWF Australia, not all nurses recognised economic hardship as part of the CHS model of care [23]. The research team did, however, encounter a higher interest among BDC services and assume this derives from the nature of their services in meeting individuals with limited resources [1] and the national guidance of the SCA encouraging preventive counselling through collaboration and outreach work [28, 29].

The next course of action for trial set-up was the General Data Protection Regulation (GDPR) agreements for data sharing [42]. The processes that followed shone a light on the degree of autonomy that Swedish municipalities hold [43], resulting in data protection and legal officers interpreting and executing the GDPR regulations differently. Owing to this process, ‘ready-to-go’ sites were put on hold for up to 4 months.

### To reach, screen and recruit participants

With the trial up and running, the team were faced with difficulties in participant recruitment. Screening was not conducted as predicted, resulting in fewer parents than expected being approached for screening. In addition, a vast majority of the services limited the screening procedure to one visit, motivated by capacity and topic insecurity. Similar to previous HWF research, the majority of nurses did not have the capacity to screen at all routine visits, forcing them to make time-based decisions [22]. In addition, there was a prevalent worry regarding how to introduce the topic of ‘economic hardship’ into the model of care [23]. However, it was identified that the routine for the 8-month visit included psychosocial factors [26], resembling the HWF screening topics. Hence, most sites therefore agreed to set an initial strategy to screen all parents at the 8-month visit with the view to expanding the initiative to further visits at a later stage.

Despite the proportion of parents screening positive showing expected rates, only a small portion of these parents expressed interest in consulting a counsellor regarding their economic hardship. This indicates low acceptability of the HWF model tested here, i.e., screening followed by referral via the research team. Upon declining, respondents were given the chance to motivate why. Content analysis of these motivations revealed both external and internal factors. A group of parents defined their worry as transient, something that would resolve upon returning to work after parental leave. They also called attention to the general uncertain times after the COVID-19 pandemic [9]. Some parents preferred to rely on a personal safety net. For example, if necessary, they would request financial means from relatives rather than seeking social benefits. This preference could be contributing to support services, such as BDC, not being perceived as being acceptable and therefore not reaching those in need.

Three key aspects regarding internal factors for declining participation in the study were knowledge, capacity and stigma. In coherence with previous research findings, experiencing hardship and inequality is no longer solely related to low-education or low-wage groups [8]. Several approached parents declined participation on the grounds of already possessing the necessary knowledge of managing finances. Other parents simply did not have the capacity nor energy to manage issues other than everyday life, as seen in the preceding literature on economic hardship and health [10]. Even though an orderly economy could improve parental well-being, the findings can be interpreted as that even just commencing a process to uncover issues around family finances is overwhelming and is therefore postponed. Previous research has shown strong relations between economic hardship and stigma [6, 7], which also came forward in the motivations for declining participation. Some respondents expressed discomfort and irritation, which could speak to the findings of the family stress model in which economic pressure is theorised to cause parental distress [4]. Overall, the qualitative findings highlight how some individuals want to ‘make do’ their own way, without the involvement of social service support.

Adding to the barrier caused by stigma was the potential burden of the questionnaire and randomisation process. Research burden has been identified as a reason for recruitment failure in trials [44], and was raised as an implementation issue in the Australian HWF project [25]. Despite efforts to reduce the burden by co-designing the survey, as well as the information given, with parents with lived experience of economic hardship, it may be the case that the process was too much of an ask for these families.

### Applying the ADePT framework

Valuable learning opportunities emerged from this pilot RCT trial, with great importance for the feasibility of a full-scale trial. Through the ADePT framework, the research team noted several problems for which potential solutions were discussed (see Table 7). Rather than relying solely on the participation of CHS, screening could take place in various settings where parents of children aged 0–5 are present, such as open preschools. The screening process could be streamlined, reducing the number of questions from five to two, and therefore reducing the burden on those performing the screening. The research process could also be streamlined to reduce the burden on participants. This could include reducing the number of questions in the survey. Enabling a direct referral to the BDC and integrating data collection into service delivery, rather than parents being required to have contact with the research team prior to being referred to BDC, would considerably reduce the burden on study participants. This approach wouldn’t be possible in a two-arm trial, as data wouldn’t be captured for the control arm, but could be achieved if the study design was amended to a single-arm non-randomised trial.

The primary outcome measure should be reconsidered, as a floor effect was evident on the Child MSD questionnaire. It’s unclear why this was the case; it could be that those recruited to the study were not experiencing the level of deprivation captured by the questionnaire or respondents answered in a socially desirable way due to the stigma of not being able to provide essentials to their child(ren). How participant income is captured should also be reviewed given the low response rate on these items. Again, it is unclear why the measurement didn’t operate as expected. It could be that the formulation of the questions was unclear, that the responding parent didn’t have an awareness of the particular type of household income queried, or that participants were actively choosing not to disclose their income.

**Table 7:** Feasibility issues and potential solutions identified using the adept framework

Problem type	Solutions
A **‘Type B’** problem in aligning service engagement **Evidence**: - Varying levels of local interest and engagement - Not all services saw their role in preventing economic hardship	**Context**: - Adjust the working model to adhere to different contexts, i.e., screening takes place in other settings where parents of 0-5-year-olds attend - Emphasise economic hardship as a health-related factor in outreach material to improve engagement among CHS
A **‘Type A’** problem in setting up data-sharing agreements **Evidence**: - The GDPR regulation was interpreted differently across services - A worry among Data Protection Officers not being in control of data	**Trial design**: - Re-design trial so participant recruitment sites do not collect data on behalf of the research team
A **‘Type B’** problem regarding the number of participants being screened **Evidence**: - The nurses had to make time-based decisions, limiting the screening to solely occur at one visit - To rely on single services made the inflow of participants go slower	**Trial design**: - Reduce the number of screening questions from five to two, to decrease the burden for nurses **Context**: - Adjust the working model to adhere to different contexts, i.e., screening takes place in other settings where parents of 0-5-year-olds attend
A **‘Type B’** problem regarding participant recruitment **Evidence**: - Upon declining participation, parents motivated decision with ‘no time or energy’ - Upon declining participation, parents motivated decision with ‘no need’	**Trial design**: Decrease burden for parents by: - Removing the randomisation and intervention/waitlist-control arms - Adjust referral pathway with a direct flow from CHS to BDC - Limit the number of survey questions **Context**: - Emphasise in outreach material the commonality of economic hardship during the toddler years, and what support BDC services can offer
A **’Type A’** problem regarding unsuitable outcome measures **Evidence**: - The primary outcome showed ‘floor effect’ - The completion rate for the questions on income was relatively low	**Trial design**: - Replace ‘Material and Social Deprivation’ with ‘Financial control’ as primary outcome measure - Reconsider how to capture participants’ income

### Methodological discussion

While the sample size for this pilot study was informed by a recognised rule of thumb based on the attributes of the main trial [32], it should still be acknowledged that the inferences that can be made from 20 participants are limited. For instance, the sample size is not sufficient to accurately inform a power calculation for the main trial; however, this was not the intention of the study. A common issue with RCTs is the representativeness of the sample [45], which may also be an issue in the present study. However, different measures were taken to promote a representative sample. Firstly, all trial documentation was co-created together with the trial advisory panel [30]. The panel was constructed to capture the different perspectives being represented in the trial: CHS nurses, counsellors at BDC and parents with lived experience of economic hardship, as well as other public sector workers and researchers working in related fields. Secondly, to address the risk of participants perceiving the study information as difficult to comprehend, considerations were made to make the written material as accessible as possible. For instance, a knowledge centre for parental cognitive disorders was consulted to adapt the language to be more accessible for parents with special needs. In addition, all included CHS centres were asked to state the main languages, besides Swedish, being represented at the centres. This was to inform the translation of the study participant information and survey, which were made available in eight additional languages as a result. This was accompanied by an offer to provide interpreter services to facilitate informed consent and data collection processes. In this way, the research team aimed to increase the reach of the trial to minority and marginalised groups.

## Conclusion

Results from the pilot RCT indicate that the screening process identified the anticipated proportion of eligible parents, but that three of the screening questions are redundant. The conversion rate was much lower than anticipated, questioning the feasibility of a full-scale RCT. BDC fidelity to protocol could be strengthened with implementation support. Finally, the primary outcome measure should be reconsidered, as well as the way in which participant income is captured. The internal pilot RCT provided valuable insights that will enhance the design of the future main trial. The findings from this Swedish trial may also contribute to the ongoing international HWF efforts.

## Electronic Supplementary Material

Below is the link to the electronic supplementary material.

Supplementary Material 1

Supplementary Material 2

## Data Availability

The data for this research consists of survey questionnaires with quantitative and qualitative data, field notes and trial management records. Raw data cannot be publicly released due to the risk of compromising participant confidentiality. For any data queries, please contact the first author (NJ).

## Abbreviations

ACCESS: Ameliorating Child poverty through Connecting Economic Services with child health Services
CHAP: Child Health and Parenting
RCT: Randomised Controlled Trial
HWF: Healthier Wealthier Families
CHS: Child Health Care System
BDC: Budget and Debt Counselling
MSD: Material and Social Deprivation
HWC: Healthier Wealthier Children
SCA: Swedish Consumer Agency
PPI: Patient and public involvement
REDCap: Research Electronic Data Capture
GDPR: General Data Protection Regulation
ADePT: A process for Decision-making after Pilot and feasibility Trials
SEK: Swedish Krona
