# Supplementary material for: Feasibility of a randomised controlled trial of Healthier Wealthier Families in Sweden: results from the Ameliorating Child poverty through Connecting Economic Services with child health Services (ACCESS) pilot study
Source: BMC Health Serv Res. 2025 Dec 1;25:1561. doi: 10.1186/s12913-025-13753-y (PMC12670747; doi:10.1186/s12913-025-13753-y)
Supplement: Supplementary file 2 — Supplementary Material 2 [file 12913_2025_13753_MOESM2_ESM.pdf]

Data Dictionary Codebook

Healthier Wealthier Families Enkätformulär, Eng (PID: 5665)

2025-03-03 14:53

Instruments		
Instrument	Form Name	Events
T1	t1	t1_arm_1
T2	t2	t2_arm_1
T3	t3	t3_arm_1

Events	
Event Name	Unique event name
T1	t1_arm_1
T2	t2_arm_1
T3	t3_arm_1

#	Variable / Field Name	Field Label<br>*Field Note*	Field Attributes (Field Type, Validation, Choices, Calculations, etc.)
**Instrument: T1 (t1)**	**Enabled as survey**		
1	[record_id]	Record ID	text
2	[introtext]	Thank you for participating in this study. This questionnaire is to get more information about your finances, your health and your family. The questions have been selected together with families who have experienced financial difficulties. It will take about 15-20 minutes to complete the questionnaire. We would be very grateful if you would take the time to answer our questions.	descriptive
3	[intro_deprivation]	Section Header:<br>Material poverty - children in the household Here are some questions about the children in the household aged between 0 and 15.	descriptive<br>Question number: 1
4	[deprivation_1]	Do children get new clothes? Not all clothes have to be newly bought. If one of the children does not receive it, answer No even if the other children in the household do	radio<br>1 Yes<br>2 No<br>3 Do not know<br>4 Do not want to answer<br>Question number: 1.1
5	[followup_1]<br>Show the field ONLY if: [deprivation_1] = '2'	If no, is it because you cannot afford it or is it for other reasons?	radio<br>1 Cannot afford<br>2 Other reasons<br>3 Do not know<br>4 Do not want to answer<br>Question number: 1.1.1
6	[deprivation_2]	Do the children have at least two pairs of shoes, including one pair of winter shoes? If one of the children does not have them, answer 'No' even if the other children in the household do	radio<br>1 Yes<br>2 No<br>3 Do not know

			4 Do not want to answer<br><br>Question number: 1.2
7	[followup_2]<br><br>Show the field ONLY if:<br>[deprivation_2] = '2'	If no, is it because you cannot afford it or is it for other reasons?	radio<br>1 Cannot afford<br>2 Other reasons<br>3 Do not know<br>4 Do not want to answer<br><br>Question number: 1.2.1
8	[deprivation_3]	Do children receive fruit and vegetables at least once a day? If one of the children does not receive it, answer 'No' even if the other children in the household do.	radio<br>1 Yes<br>2 No<br>3 Do not know<br>4 Do not want to answer<br><br>Question number: 1.3
9	[followup_3]<br><br>Show the field ONLY if:<br>[deprivation_3] = '2'	If no, is it because you cannot afford it or is it for other reasons?	radio<br>1 Cannot afford<br>2 Other reasons<br>3 Do not know<br>4 Do not want to answer<br><br>Question number: 1.3.1
10	[deprivation_4]	Do the children receive at least one meal a day of meat, chicken or fish or equivalent vegetarian food? If one of the children does not receive it, answer 'No' even if the other children in the household do.	radio<br>1 Yes<br>2 No<br>3 Do not know<br>4 Do not want to answer<br><br>Question number: 1.4
11	[followup_4]<br><br>Show the field ONLY if:<br>[deprivation_4] = '2'	If no, is it because you cannot afford it or is it for other reasons?	radio<br>1 Cannot afford<br>2 Other reasons<br>3 Do not know<br>4 Do not want to answer<br><br>Question number: 1.4.1
12	[deprivation_5]	Do you have age-appropriate books at home? School books do not count. If one of the children does not have it, answer No even if the other children in the household do	radio<br>1 Yes<br>2 No<br>3 Do not know

			4 Do not want to answer<br><br>Question number: 1.5
13	[followup_5]<br><br>Show the field ONLY if:<br>[deprivation_5] = '2'	If no, is it because you cannot afford it or is it for other reasons?	radio<br>1 Cannot afford<br>2 Other reasons<br>3 Do not know<br>4 Do not want to answer<br><br>Question number: 1.5.1
14	[deprivation_6]	Do the children have any toys or other equipment that they can use outdoors in their free time? E.g. bicycle, football, skates or sledge. If one of the children does not have it, answer 'No' even if the other children in the household do	radio<br>1 Yes<br>2 No<br>3 Do not know<br>4 Do not want to answer<br><br>Question number: 1.6
15	[followup_6]<br><br>Show the field ONLY if:<br>[deprivation_6] = '2'	If no, is it because you cannot afford it or is it for other reasons?	radio<br>1 Cannot afford<br>2 Other reasons<br>3 Do not know<br>4 Do not want to answer<br><br>Question number: 1.6.1
16	[deprivation_7]	Do the children have any toys, games or other things to occupy themselves with indoors in their free time? If one of the children does not have it, answer No even if the other children in the household do	radio<br>1 Yes<br>2 No<br>3 Do not know<br>4 Do not want to answer<br><br>Question number: 1.7
17	[followup_7]<br><br>Show the field ONLY if:<br>[deprivation_7] = '2'	If no, is it because you cannot afford it or is it for other reasons?	radio<br>1 Cannot afford<br>2 Other reasons<br>3 Do not know<br>4 Do not want to answer<br><br>Question number: 1.7.1
18	[deprivation_8]	Do children regularly participate in any leisure activities outside the home that cost money? E.g. swimming, football, dancing, scouts, theatre group, playing musical instruments, singing in choir. If one of the	radio<br>1 Yes<br>2 No<br>3 Do not know

		children does not, please indicate 'No' even if the other children in the household do.	4 Do not want to answer<br><br>Question number: 1.8
19	[followup_8]<br><br>Show the field ONLY if:<br>[deprivation_8] = '2'	If no, is it because you cannot afford it or is it for other reasons?	radio<br>1 Cannot afford<br>2 Other reasons<br>3 Do not know<br>4 Do not want to answer<br><br>Question number: 1.8.1
20	[deprivation_9]	Do your children have parties or celebrations on special days? E.g. birthday, name day. If one of the children does not have it, answer 'No' even if the other children in the household do	radio<br>1 Yes<br>2 No<br>3 Do not know<br>4 Do not want to answer<br><br>Question number: 1.9
21	[followup_9]<br><br>Show the field ONLY if:<br>[deprivation_9] = '2'	If no, is it because you cannot afford it or is it for other reasons?	radio<br>1 Cannot afford<br>2 Other reasons<br>3 Do not know<br>4 Do not want to answer<br><br>Question number: 1.9.1
22	[deprivation_10]	Do the children invite friends home to play and have a snack or dinner from time to time? If one of the children does not, please answer 'No' even if the other children in the household do	radio<br>1 Yes<br>2 No<br>3 Do not know<br>4 Do not want to answer<br><br>Question number: 1.10
23	[followup_10]<br><br>Show the field ONLY if:<br>[deprivation_10] = '2'	If no, is it because you cannot afford it or is it for other reasons?	radio<br>1 Cannot afford<br>2 Other reasons<br>3 Do not know<br>4 Do not want to answer<br><br>Question number: 1.10.1
24	[deprivation_11]	Do your children go on holiday for at least one week each year somewhere other than their home town? Please note that staying in your own holiday home or with friends or relatives also counts. If one of the children	radio<br>1 Yes<br>2 No<br>3 Do not know

		does not go on holiday, answer No even if the other children in the household do	4 Do not want to answer<br><br>Question number: 1.11
25	[followup_11]<br><br>Show the field ONLY if:<br>[deprivation_11] = '2'	If no, is it because you cannot afford it or is it for other reasons?	radio<br>1 Cannot afford<br>2 Other reasons<br>3 Do not know<br>4 Do not want to answer<br><br>Question number: 1.11.1
26	[deprivation_12]	Now a couple of questions about children up to the age of 15 who go to school. Do the children always participate in school trips or other activities organised by the school that cost money? If one of the children who goes to school does not, please answer 'No' even if the other children in the household do.	radio<br>1 Yes<br>2 No<br>3 Not applicable: The school always has a free option<br>4 Not applicable: No child in the household is in school<br>5 Do not know<br>6 Do not want to answer<br><br>Question number: 1.12
27	[followup_12]<br><br>Show the field ONLY if:<br>[deprivation_12] = '2'	If no, is it because you cannot afford it or is it for other reasons?	radio<br>1 Cannot afford<br>2 Other reasons<br>3 Do not know<br>4 Do not want to answer<br><br>Question number: 1.12.1
28	[deprivation_13]	Do the children have a suitable place at home to sit and do homework? If one of the children who goes to school does not do so, answer 'No' even if the other children in the household do.	radio<br>1 Yes<br>2 No<br>3 Not applicable: No child in the household is in school<br>4 Do not know<br>5 Do not want to answer<br><br>Question number: 1.13
29	[followup_13]<br><br>Show the field ONLY if:<br>[deprivation_13] = '2'	If no, is it because you cannot afford it or is it for other reasons?	radio<br>1 Cannot afford<br>2 Other reasons<br>3 Do not know<br>4 Do not want to answer

			Question number: 1.13.1
30	[deprivation_14]	Here are two questions about you. Are you a member of the unemployment fund (A-kassa)?	radio<br>1 Yes<br>2 No<br>3 Do not know<br>4 Do not want to answer<br><br>Question number: 1.14
31	[followup_14]<br><br>Show the field ONLY if: [deprivation_14] = '2'	If no, is it because you cannot afford it or is it for other reasons?	radio<br>1 Cannot afford<br>2 Other reasons<br>3 Do not know<br>4 Do not want to answer<br><br>Question number: 1.14.1
32	[deprivation_15]	Does your home have home insurance?	radio<br>1 Yes<br>2 No<br>3 Do not know<br>4 Do not want to answer<br><br>Question number: 1.15
33	[followup_15]<br><br>Show the field ONLY if: [deprivation_15] = '2'	If no, is it because you cannot afford it or is it for other reasons?	radio<br>1 Cannot afford<br>2 Other reasons<br>3 Do not know<br>4 Do not want to answer<br><br>Question number: 1.15.1
34	[intro_fincontrol]	Section Header: *10% 13%*<br><br>Financial control In this section, we want to ask you about your perception of your financial control. On a scale of 1-5, where 1 is the lowest possible and 5 is the highest possible, please answer how much or little you agree with each statement.	descriptive<br>Question number: 2
35	[fincontrol_1]	My financial situation is largely beyond my control	radio (Matrix)<br>1 Disagrees<br>2 2<br>3 3<br>4 4<br>5 Agrees

			Question number: 2.1
36	[fincontrol_2]	I have a good insight in my household's finances	radio (Matrix)<br>1 Disagrees<br>2 2<br>3 3<br>4 4<br>5 Agrees<br><br>Question number: 2.2
37	[fincontrol_3]	It is possible for me to set long-term financial goals and work to meet them	radio (Matrix)<br>1 Disagrees<br>2 2<br>3 3<br>4 4<br>5 Agrees<br><br>Question number: 2.3
38	[fincontrol_4]	It is possible for me to have good control over my spending	radio (Matrix)<br>1 Disagrees<br>2 2<br>3 3<br>4 4<br>5 Agrees<br><br>Question number: 2.4
39	[fincontrol_5]	It is possible for me to have good control over my income	radio (Matrix)<br>1 Disagrees<br>2 2<br>3 3<br>4 4<br>5 Agrees<br><br>Question number: 2.5
40	[fincontrol_6]	I am able to save part of my income each month	radio (Matrix)<br>1 Disagrees<br>2 2<br>3 3<br>4 4<br>5 Agrees<br><br>Question number: 2.6

<table>
<tr><td>41</td><td>[fincontrol_7]</td><td>I worry daily about money problems</td><td>radio (Matrix)<table><tr><td>1</td><td>Disagrees</td></tr><tr><td>2</td><td>2</td></tr><tr><td>3</td><td>3</td></tr><tr><td>4</td><td>4</td></tr><tr><td>5</td><td>Agrees</td></tr></table>Question number: 2.7</td></tr>
<tr><td>42</td><td>[fincontrol_8]</td><td>I know what my rights are as a consumer, such as complaints</td><td>radio (Matrix)<table><tr><td>1</td><td>Disagrees</td></tr><tr><td>2</td><td>2</td></tr><tr><td>3</td><td>3</td></tr><tr><td>4</td><td>4</td></tr><tr><td>5</td><td>Agrees</td></tr></table>Question number: 2.8</td></tr>
<tr><td>43</td><td>[fincontrol_9]</td><td>I know what allowances and benefits I am entitled to</td><td>radio (Matrix)<table><tr><td>1</td><td>Disagrees</td></tr><tr><td>2</td><td>2</td></tr><tr><td>3</td><td>3</td></tr><tr><td>4</td><td>4</td></tr><tr><td>5</td><td>Agrees</td></tr></table>Question number: 2.9</td></tr>
<tr><td>44</td><td>[intro_income]</td><td>Section Header: 20% 21%
Income Tick the options that are an income for your family</td><td>descriptive
Question number: 3</td></tr>
<tr><td>45</td><td>[income_1]</td><td>Wage</td><td>radio (Matrix)<table><tr><td>1</td><td>Yes</td></tr><tr><td>2</td><td>Do not know</td></tr></table>Question number: 3.1</td></tr>
<tr><td>46</td><td>[income_2]</td><td>Pension</td><td>radio (Matrix)<table><tr><td>1</td><td>Yes</td></tr><tr><td>2</td><td>Do not know</td></tr></table>Question number: 3.2</td></tr>
<tr><td>47</td><td>[income_3]</td><td>Activity allowance</td><td>radio (Matrix)<table><tr><td>1</td><td>Yes</td></tr><tr><td>2</td><td>Do not know</td></tr></table>Question number: 3.3</td></tr>
<tr><td>48</td><td>[income_4]</td><td>Sickness allowance</td><td>radio (Matrix)</td></tr>
</table>

			<table><tr><td>1</td><td>Yes</td></tr><tr><td>2</td><td>Do not know</td></tr></table><br>Question number: 3.4
49	[income_5]	Labour market support (Unemployment benefit)	radio (Matrix)<br><table><tr><td>1</td><td>Yes</td></tr><tr><td>2</td><td>Do not know</td></tr></table><br>Question number: 3.5
50	[income_6]	Study allowance	radio (Matrix)<br><table><tr><td>1</td><td>Yes</td></tr><tr><td>2</td><td>Do not know</td></tr></table><br>Question number: 3.6
51	[income_7]	Study loan	radio (Matrix)<br><table><tr><td>1</td><td>Yes</td></tr><tr><td>2</td><td>Do not know</td></tr></table><br>Question number: 3.7
52	[income_8]	Housing benefit	radio (Matrix)<br><table><tr><td>1</td><td>Yes</td></tr><tr><td>2</td><td>Do not know</td></tr></table><br>Question number: 3.8
53	[income_9]	Housing benefit (addition)	radio (Matrix)<br><table><tr><td>1</td><td>Yes</td></tr><tr><td>2</td><td>Do not know</td></tr></table><br>Question number: 3.9
54	[income_10]	Income support	radio (Matrix)<br><table><tr><td>1</td><td>Yes</td></tr><tr><td>2</td><td>Do not know</td></tr></table><br>Question number: 3.10
55	[income_11]	Assistance allowance for adults	radio (Matrix)<br><table><tr><td>1</td><td>Yes</td></tr><tr><td>2</td><td>Do not know</td></tr></table><br>Question number: 3.11
56	[income_12]	Disability benefit	radio (Matrix)<br><table><tr><td>1</td><td>Yes</td></tr><tr><td>2</td><td>Do not know</td></tr></table><br>Question number: 3.12

57	[income_13]	Additional costs allowance for adults	radio (Matrix)<br>1 Yes<br>2 Do not know<br><br>Question number: 3.13
58	[income_14]	Specialised high-risk support	radio (Matrix)<br>1 Yes<br>2 Do not know<br><br>Question number: 3.14
59	[income_15]	Car allowance for adults	radio (Matrix)<br>1 Yes<br>2 Do not know<br><br>Question number: 3.15
60	[income_16]	Parental benefit	radio (Matrix)<br>1 Yes<br>2 Do not know<br><br>Question number: 3.16
61	[income_17]	Pregnancy benefit	radio (Matrix)<br>1 Yes<br>2 Do not know<br><br>Question number: 3.17
62	[income_18]	Maintenance support	radio (Matrix)<br>1 Yes<br>2 Do not know<br><br>Question number: 3.18
63	[income_19]	Alimony payments	radio (Matrix)<br>1 Yes<br>2 Do not know<br><br>Question number: 3.19
64	[income_20]	Child benefit	radio (Matrix)<br>1 Yes<br>2 Do not know<br><br>Question number: 3.20
65	[income_21]	Multiple child allowance	radio (Matrix)<br>1 Yes<br>2 Do not know

			Question number: 3.21
66	[income_22]	Care allowance	radio (Matrix)<br>1 Yes<br>2 Do not know<br><br>Question number: 3.22
67	[income_23]	Carer's allowance	radio (Matrix)<br>1 Yes<br>2 Do not know<br><br>Question number: 3.23
68	[income_24]	Additional costs for children	radio (Matrix)<br>1 Yes<br>2 Do not know<br><br>Question number: 3.24
69	[income_25]	Assistance allowance for children	radio (Matrix)<br>1 Yes<br>2 Do not know<br><br>Question number: 3.25
70	[income_26]	Car allowance for children	radio (Matrix)<br>1 Yes<br>2 Do not know<br><br>Question number: 3.26
71	[income_other]	If other, write your answer here	notes<br>Question number: 3.27
72	[intro_covid]	Changed economic situation due to Covid-19.	descriptive
73	[covid_1]	New debts and/or credits?	radio<br>1 Yes<br>2 Do not know<br>3 Other<br><br>Question number: 3.28
74	[covid_other]<br><br>Show the field ONLY if:<br>[covid_1] = '3'	If other, write your answer here:	notes<br>Question number: 3.28.1
75	[income_earnings]	Approximately how much is the household income before tax in a typical month? Include any benefits, allowances, supplements and allowances	dropdown<br>1 0 - 2499<br>2 2500 - 4999

			3 5000 - 7499<br>4 7500 - 9999<br>5 10 000 - 12 499<br>6 12 500 - 14 999<br>7 15 000 - 17 499<br>8 17 500 - 19 999<br>9 20 000 - 22 499<br>10 22 500 - 24 999<br>11 25 000 - 27 499<br>12 27 500 - 29 999<br>13 30 000 - 32 499<br>14 Over 32 500<br>15 Do not know<br>16 Do not want to answer<br><br>Question number: 3.29
76	[income_expenses]	Approximately how much do you pay in fixed costs each month, i.e. approximately how much are the costs you have to pay each month, such as rent and bills?	notes<br>Question number: 3.30
77	[care_cost]	Does someone in your household have a care need that requires recurring costs such as medication or special diets?	radio<br>1 Yes<br>2 No<br><br>Question number: 3.31
78	[careneed_spec]<br><br>Show the field ONLY if:<br>[care_cost] = '1'	If yes, please specify:	notes<br>Question number: 3.31.3
79	[intro_change]	Section Header: *45% 47%*<br>Change In this section we want to ask you about your thoughts on change. On a scale of 1-10, where 1 is the lowest possible and 10 is the highest possible, how much or how little do you agree with this statement?	descriptive<br>Question number: 4
80	[change_1]	How ready are you to change your financial situation? If you are not ready to make a change at all, choose 1. If you are already working hard to make a change, choose 10.	radio (Matrix)<br>1 1<br>2 2<br>3 3<br>4 4<br>5 5<br>6 6<br>7 7

<table>
<tr><td></td><td></td><td></td><td><table><tr><td>8</td><td>8</td></tr><tr><td>9</td><td>9</td></tr><tr><td>10</td><td>10</td></tr></table>Question number: 4.1</td></tr>
<tr><td>81</td><td>[intro_goal]</td><td>Goal setting These questions are about setting goals to change your financial situation.</td><td>descriptive
Question number: 5</td></tr>
<tr><td>82</td><td>[goal_1]</td><td>If you were to set a personal goal to change your financial situation over the next 3 months, what would it be?</td><td>notes
Question number: 5.1</td></tr>
<tr><td>83</td><td>[intro_finknowledge]</td><td>Section Header: Now 50%, i.e. half of the survey, has been completed 50%
Financial knowledge In this section we want to ask you about your financial knowledge. The first part is about where to turn for help.</td><td>descriptive
Question number: 6</td></tr>
<tr><td>84</td><td>[finknowledge_1]</td><td>Where would you turn for help if you need better control over your finances, to see where your money is going and get tips to make it last longer?</td><td>radio<table><tr><td>1</td><td>Bank/loan company</td></tr><tr><td>2</td><td>Budget and debt counselling in the municipality</td></tr><tr><td>3</td><td>Consumer Agency</td></tr><tr><td>4</td><td>Consumer agencies</td></tr><tr><td>5</td><td>Voluntary organisations</td></tr><tr><td>6</td><td>The Enforcement Authority (Kronofogden)</td></tr><tr><td>7</td><td>Not taking help from anyone</td></tr><tr><td>8</td><td>Other</td></tr><tr><td>9</td><td>Do not know / Do not want to answer</td></tr></table>Question number: 6.1</td></tr>
<tr><td>85</td><td>[finknowledge_2]</td><td>Where would you go for help if you have debts you can't pay and need help to find out what you can do?</td><td>radio<table><tr><td>1</td><td>Bank/loan company</td></tr><tr><td>2</td><td>Budget and debt counselling in the municipality</td></tr><tr><td>3</td><td>Consumer Agency</td></tr><tr><td>4</td><td>Consumer agencies</td></tr><tr><td>5</td><td>Voluntary organisations</td></tr><tr><td>6</td><td>The Enforcement Authority (Kronofogden)</td></tr><tr><td>7</td><td>Not taking help from anyone</td></tr><tr><td>8</td><td>Other</td></tr><tr><td>9</td><td>Do not know / Do not want to answer</td></tr></table></td></tr>
</table>

			Question number: 6.2
86	[finknowledge_3]	Where would you turn for help if you have very large debts that you can't pay and need help with debt management?	radio<br>1 Bank/loan company<br>2 Budget and debt counselling in the municipality<br>3 Consumer Agency<br>4 Consumer agencies<br>5 Voluntary organisations<br>6 The Enforcement Authority (Kronofogden)<br>7 Not taking help from anyone<br>8 Other<br>9 Do not know / Do not want to answer<br><br>Question number: 6.3
87	[intro_ecoknowledge]	These questions are about your general financial knowledge.	descriptive<br>Question number: 7
88	[ecoknowledge_1]	Do you know what happens if you receive an invoice: when is the latest you have to pay it?	radio<br>1 In 10 days<br>2 In 15 days<br>3 In 30 days<br>4 By the date indicated on the invoice<br>5 Do not know<br><br>Question number: 7.1
89	[ecoknowledge_2]	What is an amortisation payment?	radio<br>1 One type of interest rate<br>2 An instalment<br>3 One type of fee<br>4 A type of tax<br>5 Do not know<br><br>Custom alignment: LH<br>Question number: 7.2
90	[ecoknowledge_3]	On which of the following occasions do you have a payment default?	radio<br>1 When I receive a debt collection letter from a debt collection agency<br>2 When the unpaid invoice is sent to the Enforcement Authority

			3 When I receive a decision, a so-called ruling, from Kronofogden<br>4 When a company sends a second reminder with a debt collection warning<br>5 Do not know<br><br>Question number: 7.3
91	[intro_demo]	Section Header: *55% 55%*<br>Demographics These questions are about you and your household	descriptive<br>Question number: 8
92	[demo_1]	How many children live in your home?	radio<br>1 1<br>2 2<br>3 3<br>4 4<br>5 5<br>6 6<br>7 More than 6<br><br>Question number: 8.1
93	[demo_2]<br><br>Show the field ONLY if:<br>[demo_1] = '1' or [demo_1] = '2' or [demo_1] = '3' or [demo_1] = '4' or [demo_1] = '5' or [demo_1] = '6' or [demo_1] = '7'	How old are the children living in your home? (State age)	descriptive<br>Question number: 8.2
94	[demo_2_1]<br><br>Show the field ONLY if:<br>[demo_1] = '1' or [demo_1] = '2' or [demo_1] = '3' or [demo_1] = '4' or [demo_1] = '5' or [demo_1] = '6' or [demo_1] = '7'	Child 1	text<br>Question number: 8.2.1
95	[demo_2_2]<br><br>Show the field ONLY if:<br>[demo_1] = '2' or [demo_1] = '3' or [demo_1] = '4' or [demo_1] = '5' or [demo_1] = '6' or [demo_1] = '7'	Child 2	text<br>Question number: 8.2.2

96	[demo_2_3]<br><br>Show the field ONLY if:<br>[demo_1] = '3' or [demo_1] = '4' or [demo_1] = '5' or [demo_1] = '6' or [demo_1] = '7'	Child 3	text<br>Question number: 8.2.3
97	[demo_2_4]<br><br>Show the field ONLY if:<br>[demo_1] = '4' or [demo_1] = '5' or [demo_1] = '6' or [demo_1] = '7'	Child 4	text<br>Question number: 8.2.4
98	[demo_2_5]<br><br>Show the field ONLY if:<br>[demo_1] = '5' or [demo_1] = '6' or [demo_1] = '7'	Child 5	text<br>Question number: 8.2.5
99	[demo_2_6]<br><br>Show the field ONLY if:<br>[demo_1] = '6' or [demo_1] = '7'	Child 6	text<br>Question number: 8.2.6
100	[demo_2_7]<br><br>Show the field ONLY if:<br>[demo_1] = '7'	If there are more than 6 children in your family, write the number and age here:	text<br>Question number: 8.2.7
101	[demo_partner]	Do you have a partner right now?	radio<br>1 Yes<br>2 No<br><br>Question number: 8.3
102	[demo_partner_2]<br><br>Show the field ONLY if:<br>[demo_partner] = '1'	Does your partner live with you?	radio<br>1 Yes<br>2 No<br><br>Question number: 8.4
103	[demo_care]	Does someone in your home have a disability or a need for special care or diet?	radio<br>1 Yes<br>2 No<br><br>Question number: 8.5
104	[demo_origin]	Where were you born?	radio<br>1 In Sweden

			2 In another Nordic country<br>3 In another European country outside of Nordic<br>4 In non-European country<br><br>Question number: 8.6
105	[demo_language]	What language do you speak at home?	radio<br>1 Swedish<br>2 Other<br><br>Question number: 8.7
106	[demo_language_2]<br><br>Show the field ONLY if:<br>[demo_language] = '2'	Write your answer for other:	text<br>Question number: 8.7.1
107	[demo_education]	What is the highest level of education you have completed?	radio<br>1 Primary school or equivalent<br>2 Secondary school diploma or equivalent<br>3 University or college education less than 3 years<br>4 University or college education of 3 years or more<br>5 Other<br><br>Question number: 8.8
108	[demo_education_2]<br><br>Show the field ONLY if:<br>[demo_education] = '5'	Write your answer for other:	text<br>Question number: 8.8.1
109	[intro_demo_work]	About work	descriptive<br>Question number: 9
110	[demo_work1]	Employment	radio<br>1 Employed<br>2 Self-employed<br>3 Student<br>4 Unemployed<br>5 Unemployed in employment<br>6 On sick leave<br>7 Other<br><br>Question number: 9.1

111	[demo_work2]<br><br>Show the field ONLY if:<br>[demo_work1] = '6'	What percentage are you on sick leave?	text<br>Question number: 9.2
112	[demo_work_1_1]<br><br>Show the field ONLY if:<br>[demo_work1] = '7'	Write your answer for other:	notes<br>Custom alignment: RH<br>Question number: 9.1.1
113	[demo_work_2]<br><br>Show the field ONLY if:<br>[demo_work1] = '2' or [demo_work1] = '1'	Do you work full-time, part-time or as needed?	radio<br>1 Full-time<br>2 Part-time (Less than 35 hours/week)<br>3 If needed<br>4 Other<br><br>Question number: 9.3
114	[demo_work_2_1]<br><br>Show the field ONLY if:<br>[demo_work_2] = '4'	Write your answer for other:	notes<br>Question number: 9.3.1
115	[demo_work_3]<br><br>Show the field ONLY if:<br>[demo_work1] = '1' or [demo_work1] = '2'	Would you like to work more than you do now?	radio<br>1 Yes<br>2 No<br>3 Other<br><br>Question number: 9.4
116	[demo_work_3_1]<br><br>Show the field ONLY if:<br>[demo_work_3] = '3'	Write your answer for other:	notes<br>Question number: 9.4.1
117	[demo_work_4]	Have you looked for a job at any time in the last 4 weeks?	radio<br>1 Yes<br>2 No<br>3 Other<br><br>Question number: 9.5
118	[demo_work_4_1]<br><br>Show the field ONLY if:<br>[demo_work_4] = '3'	Write your answer for other:	notes<br>Question number: 9.5.1
119	[demo_housing]	About Housing	descriptive<br>Question number: 10
120	[demo_housing_1]	How do you currently live?	radio<br>1 First-hand rental apartment

			2 Second-hand rented flat (third-hand etc)<br>3 Residential property, Apartment<br>4 Own villa/townhouse<br>5 Other accommodation<br><br>Question number: 10.1
121	[demo_housing_1_1]<br><br>Show the field ONL Y if:<br>[demo_housing_1] = '5'	Write your answer for other:	notes<br>Question number: 10.1.1
122	[intro_ghq12]	Section Header: *70% 66%*<br>GHQ12 These questions are about how you have felt and how you have been feeling in the last few weeks.	descriptive<br>Question number: 11
123	[ghq12_1]	In recent weeks, have you been able to concentrate on everything you have been doing?	radio<br>1 Better than usual<br>2 As usual<br>3 Worse than usual<br>4 Much worse than usual<br><br>Question number: 11.1
124	[ghq12_2]	Have you had trouble sleeping in recent weeks due to anxiety?	radio<br>1 Not at all<br>2 No more than usual<br>3 More than usual<br>4 Much more than usual<br><br>Question number: 11.2
125	[ghq12_3]	In the last few weeks, have you felt that you have made a difference?	radio<br>1 More than usual<br>2 As usual<br>3 Less than usual<br>4 much less than usual<br><br>Question number: 11.3
126	[ghq12_4]	Have you been able to make decisions on various issues in recent weeks?	radio<br>1 Better than usual<br>2 As usual<br>3 Worse than usual<br>4 Much worse than usual<br><br>Question number: 11.4

127	[ghq12_5]	Have you constantly felt tense in recent weeks?	radio<br>1 Not at all<br>2 No more than usual<br>3 More than usual<br>4 Much more than usual<br><br>Question number: 11.5
128	[ghq12_6]	In the last few weeks, have you felt that you could not cope with your problems?	radio<br>1 Not at all<br>2 No more than usual<br>3 More than usual<br>4 Much more than usual<br><br>Question number: 11.6
129	[ghq12_7]	In the last few weeks, have you felt that you could appreciate what you did during the day?	radio<br>1 More than usual<br>2 As usual<br>3 Less than usual<br>4 Much less than usual<br><br>Question number: 11.7
130	[ghq12_8]	Have you been able to deal with your problems in recent weeks?	radio<br>1 Better than usual<br>2 As usual<br>3 Worse than usual<br>4 Much worse than usual<br><br>Question number: 11.8
131	[ghq12_9]	Have you been constantly feeling unhappy and depressed in recent weeks?	radio<br>1 Not at all<br>2 No more than usual<br>3 More than usual<br>4 Much more than usual<br><br>Question number: 11.9
132	[ghq12_10]	Have you lost faith in yourself in recent weeks?	radio<br>1 Not at all<br>2 No more than usual<br>3 More than usual<br>4 Much more than usual<br><br>Question number: 11.10

133	[ghq12_11]	Have you thought you were useless in recent weeks?	radio<br>1 Not at all<br>2 No more than usual<br>3 More than usual<br>4 Much more than usual<br><br>Question number: 11.11
134	[ghq12_12]	Have you felt generally happy in the last few weeks?	radio<br>1 Not at all<br>2 No more than usual<br>3 More than usual<br>4 Much more than usual<br><br>Question number: 11.12
135	[intro_stigma]	Section Header: *80% 77%*<br>Stigma This section is about feelings and experiences of financial difficulties. Think back to your feelings over the last 6 months. On a scale of 1-5, where 1 is the lowest possible and 5 is the highest possible, answer how much or little you agree with each statement	descriptive<br>Question number: 12
136	[stigma_1]	I feel odd or abnormal because of my financial situation	radio (Matrix)<br>1 Do not agree<br>2 2<br>3 3<br>4 4<br>5 Agree<br><br>Question number: 12.1
137	[stigma_2]	There have been times when I have been ashamed of my financial situation	radio (Matrix)<br>1 Do not agree<br>2 2<br>3 3<br>4 4<br>5 Agree<br><br>Question number: 12.2
138	[stigma_3]	I never feel self-conscious when I am in a public place	radio (Matrix)<br>1 Do not agree<br>2 2<br>3 3<br>4 4

			5 Agree<br><br>Question number: 12.3
139	[stigma_4]	I am never ashamed of my financial situation	radio (Matrix)<br>1 Do not agree<br>2 2<br>3 3<br>4 4<br>5 Agree<br><br>Question number: 12.4
140	[stigma_5]	I feel that people look down on me because of my financial situation	radio (Matrix)<br>1 Do not agree<br>2 2<br>3 3<br>4 4<br>5 Agree<br><br>Question number: 12.5
141	[stigma_6]	People treat me differently because of my economic situation	radio (Matrix)<br>1 Do not agree<br>2 2<br>3 3<br>4 4<br>5 Agree<br><br>Question number: 12.6
142	[stigma_7]	I have experienced people saying negative or mean things about me behind my back because of my financial situation	radio (Matrix)<br>1 Do not agree<br>2 2<br>3 3<br>4 4<br>5 Agree<br><br>Question number: 12.7
143	[stigma_8]	I have been excluded from work, school and/or family life because of my financial situation	radio (Matrix)<br>1 Do not agree<br>2 2<br>3 3<br>4 4<br>5 Agree

			Question number: 12.8
144	[intro_godaar]	Section Header: *85% 84%*<br>CALY Instructions: Fill in each question. Tick only one option. The questions are statements that apply to your current situation. Tick the option that is most true.	descriptive<br>Question number: 13
145	[godaar_1]	I have a good general state of health (mental and physical) that almost always (say at least 95% of all days) allows me to work or do what I want	radio (Matrix)<br>1 Fully agree<br>2 Partially agree<br>3 Disagree<br>Question number: 13.1
146	[godaar_2]	I have access to close relationships (family, friends or acquaintances) who give me advice and support when I need it	radio (Matrix)<br>1 Fully agree<br>2 Partially agree<br>3 Disagree<br>Question number: 13.2
147	[godaar_3]	I have a financial situation (salary, other income or savings) that always allows me to have a permanent home and most of the time (at least 8 times out of 10) allows me to buy what I think I need	radio (Matrix)<br>1 Fully agree<br>2 Partially agree<br>3 Disagree<br>Question number: 13.3
148	[godaar_4]	I have a job or other occupation (study, training, homework, caring for a relative, etc.) that I am mostly satisfied with. In the last five years, I have been satisfied with what I have been doing at least 75% of the time	radio (Matrix)<br>1 Fully agree<br>2 Partially agree<br>3 Disagree<br>Question number: 13.4
149	[godaar_5]	I have a sense of security that the risk of violence or other criminality does not affect me in my work or in my free time	radio (Matrix)<br>1 Fully agree<br>2 Partially agree<br>3 Disagree<br>Question number: 13.5
150	[godaar_6]	I trust that our rights as citizens, and our active participation in the democratic system, make it possible to influence our common living conditions	radio (Matrix)<br>1 Fully agree<br>2 Partially agree<br>3 Disagree<br>Question number: 13.6

151	[intro_cont]	Section Header: *90% 89%*<br>Consumption of resources These questions are about your contacts with health services in the last 3 months. In the last 3 months, have you had contact with:	descriptive<br>Question number: 14
152	[hcare1]	Doctor in health centre/similar	radio<br>1 Yes<br>2 No<br>Question number: 14.1
153	[hcare1_1]<br>Show the field ONLY if:<br>[hcare1] = '1'	Number of times	text<br>Question number: 14.1.1
154	[hcare2]	Doctors in hospitals	radio<br>1 Yes<br>2 No<br>Question number: 14.2
155	[hcare2_1]<br>Show the field ONLY if:<br>[hcare2] = '1'	Number of times	text<br>Question number: 14.2.1
156	[hcare3]	Psychologist	radio<br>1 Yes<br>2 No<br>Question number: 14.3
157	[hcare3_1]<br>Show the field ONLY if:<br>[hcare3] = '1'	Number of times	text<br>Question number: 14.3.1
158	[hcare4]	Curator	radio<br>1 Yes<br>2 No<br>Question number: 14.4
159	[hcare4_1]<br>Show the field ONLY if:<br>[hcare4] = '1'	Number of times	text<br>Question number: 14.4.1
160	[hcare5]	Nurse	radio<br>1 Yes<br>2 No

			Question number: 14.5
161	[hcare5_1]<br><br>Show the field ONLY if:<br>[hcare5] = '1'	Number of times	text<br>Question number: 14.5.1
162	[hcare6]	Hospitalised	radio<br>1 Yes<br>2 No<br><br>Question number: 14.6
163	[hcare6_1]<br><br>Show the field ONLY if:<br>[hcare6] = '1'	Number of times	text<br>Question number: 14.6.1
164	[hcare7]	Participated in group activities	radio<br>1 Yes<br>2 No<br><br>Question number: 14.7
165	[hcare7_1]<br><br>Show the field ONLY if:<br>[hcare7] = '1'	Number of times	text<br>Question number: 14.7.1
166	[intro_interv]	Municipal interventions These questions are about your contacts with the municipality in the last three months In the last 3 months, have you had contact with:	descriptive<br>Question number: 15
167	[interv_1]	Housing support	radio (Matrix)<br>1 Yes<br>2 Do not know<br><br>Question number: 15.1
168	[interv_2]	Specialised housing	radio (Matrix)<br>1 Yes<br>2 Do not know<br><br>Question number: 15.2
169	[interv_3]	Good man	radio (Matrix)<br>1 Yes<br>2 Do not know<br><br>Question number: 15.3
170	[interv_4]	Personal representative	radio (Matrix)<br>1 Yes

			2 Do not know<br><br>Question number: 15.4
171	[interv_5]	Contact person	radio (Matrix)<br>1 Yes<br>2 Do not know<br><br>Question number: 15.5
172	[interv_6]	Meeting centre/employment	radio (Matrix)<br>1 Yes<br>2 Do not know<br><br>Question number: 15.6
173	[intro_final]	Section Header: *100% You have now completed the survey! 100%*<br><br>Thank you for completing this survey! To finish and submit the survey, click on the button that says 'Submit'	descriptive
174	[t1_complete]	Section Header: *Form Status*<br><br>Complete?	dropdown<br>0 Incomplete<br>1 Unverified<br>2 Complete
**Instrument: T2 (t2)**		**Enabled as survey**	
175	[introtext_t2]	Tack för att du deltar i denna studie. Detta frågeformulär är för att vi ska få mer information om din ekonomi, din hälsa och din familj. Vissa frågor kan vara lika varandra. Svara på frågorna så gott du kan.	descriptive
176	[t2_fin_counselling]	Har du under den senaste månaden använt ekonomiska rådgivningstjänster? (Med det menar vi möte med en budget- och skuldrådgivare för att få hjälp med din ekonomi)	radio<br>1 Ja<br>2 Nej<br><br>Question number: 0,1
177	[intro_deprivation_t2]	Section Header:<br><br>Materiell fattigdom - barn i hushållet Nu kommer några frågor om de barn i hushållet som är mellan 0 och 15 år.	descriptive<br>Question number: 1
178	[deprivation_1_t2]	Får barnen nya kläder? Alla kläder behöver inte vara nyinköpta. Om ett av barnen inte får det ska Nej-svar anges även om de övriga barnen i hushållet får det	radio<br>1 Ja<br>2 Nej<br>3 Vet ej<br>4 Vill ej svara<br><br>Question number: 1.1
